# Genome sequence of *Ensifer medicae* Di28; an effective N_2_-fixing microsymbiont of *Medicago murex and M. polymorpha*

**DOI:** 10.1186/1944-3277-9-4

**Published:** 2014-12-08

**Authors:** Giovanni Garau, Jason Terpolilli, Yvette Hill, Rui Tian, John Howieson, Lambert Bräu, Lynne Goodwin, James Han, TBK Reddy, Marcel Huntemann, Amrita Pati, Tanja Woyke, Konstantinos Mavromatis, Victor Markowitz, Natalia Ivanova, Nikos Kyrpides, Wayne Reeve

**Affiliations:** 1 Dipartimento di Agraria, S.T.A.A., University of Sassari, Sassari, Italy; 2 Centre for Rhizobium Studies, Murdoch University, Murdoch, WA, Australia; 3 School of Life and Environmental Sciences, Deakin University, Deakin, VIC, Australia; 4 Los Alamos National Laboratory, Bioscience Division, Los Alamos, NM, USA; 5 DOE Joint Genome Institute, Walnut Creek, CA, USA; 6 Biological Data Management and Technology Center, Lawrence Berkeley National Laboratory, Berkeley, CA, USA; 7 Department of Biological Sciences, King Abdulaziz University, Jeddah, Saudi Arabia

**Keywords:** Root-nodule bacteria, Nitrogen fixation, Rhizobia, *Alphaproteobacteria*

## Abstract

*Ensifer medicae* Di28 is an aerobic, motile, Gram-negative, non-spore-forming rod that can exist as a soil saprophyte or as a legume microsymbiont of *Medicago* spp. Di28 was isolated in 1998 from a nodule recovered from the roots of *M. polymorpha* growing in the south east of Sardinia (Italy). Di28 is an effective microsymbiont of the annual forage legumes *M. polymorpha* and *M. murex* and is capable of establishing a partially effective symbiotic association with the perennial *M. sativa.* Here we describe the features of *E. medicae* Di28, together with genome sequence information and its annotation. The 6,553,624 bp standard draft genome is arranged into 104 scaffolds of 104 contigs containing 6,394 protein-coding genes and 75 RNA-only encoding genes. This rhizobial genome is one of 100 sequenced as part of the DOE Joint Genome Institute 2010 Genomic Encyclopedia for Bacteria and Archaea-Root Nodule Bacteria (GEBA-RNB) project.

## Introduction

Legumes are key components of sustainable agricultural systems owing to their ability to form nitrogen (N_2_)-fixing symbioses with specific soil bacteria referred to as rhizobia (or root nodule bacteria). These rhizobia are housed within legume root nodules, where they receive a source of carbon from the legume and in return supply the host with reduced nitrogen (N) in the form of ammonia [1]. The provision of this bioavailable N to the host fuels legume growth and development without the requirement for supplementation with industrially synthesized N-based fertilizers. Furthermore, some of this biologically fixed N remains in the soil after plant harvest or senescence, resulting in an increase in soil fertility. Growing legumes in rotation with a cereal crop or as a source of forage or fodder is therefore an environmentally sustainable way of improving soil fertility and boosting agricultural productivity [2].

The legume genus *Medicago* is of prime importance globally as a source of forage or fodder. The perennial *M. sativa* (alfalfa or lucerne) is the most widely cultivated member of this genus, with over 35 million hectares grown annually. Other important species include the annuals *M. polymorpha* (burr medic), *M. truncatula*, (barrel medic) and *M. murex*[2-4]. In order to maximise the agronomic success of these forage legumes, it is imperative that they are well-matched with an effective N_2_-fixing microsymbiont [5,6]. While *Ensifer meliloti* and *E. medicae* are two species of rhizobia both able to nodulate and fix N_2_ with *Medicago* spp., differences exist between these species with regard to their host range and effectiveness. Specifically, *E. medicae* is an effective N_2_-fixing symbiont of the acid tolerant annual *Medicago* spp. (e.g. *M. polymorpha*, *M. murex* and *M. arabica*), whereas *E. meliloti* adapted to nodulate and fix N_2_ with the neutral or slightly alkaline-favoring *M. truncatula*, *M. littoralis* and *M. tornata*[7-9].

The strain *E. medicae* Di28 was isolated in 1998 from a nodule collected from *M. polymorpha* growing in the south east of Sardinia (Italy) [10]. In common with many *E. medicae* strains, *E. medicae* Di28 is only moderately effective as a microsymbiont with *M. sativa*[8]. However, Di28 is capable of effective N_2_ fixation with *M. murex* and *M. polymorpha*[8]. Therefore, this strain is a valuable resource in improving our understanding of the genetic determinants of highly efficient N_2_-fixing symbioses and host range, which would complement information already gained from the sequencing of the genomes of other *Medicago*-nodulating microsymbionts [11-13]. Here we present a summary classification and a set of general features for this microsymbiont together with a description of its genome sequence and annotation.

## Organism information

### Classification and general features

**Figure 1:**
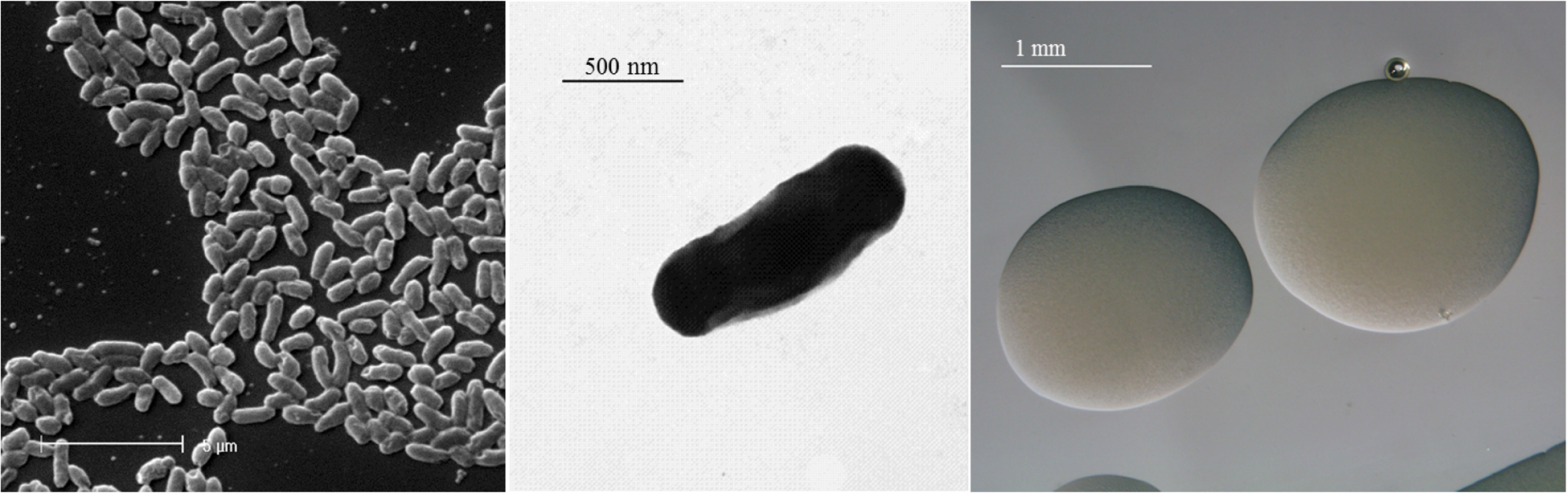
**Images of *Ensifer medicae *** **Di28 using scanning (Left) and transmission (Center) electron microscopy and the appearance of colony morphology on ½LA solid medium (Right).**

*E. medicae* Di28 is a motile, Gram-negative rod (Figure 1 Left and Center) in the order *Rhizobiales* of the class *Alphaproteobacteria*. It is fast growing, forming colonies within 3–4 days when grown on half strength Lupin Agar (½LA) [14] at 28°C. Colonies on ½LA are white-opaque, slightly domed and moderately mucoid with smooth margins (Figure 1 Right).

**Figure 2:**
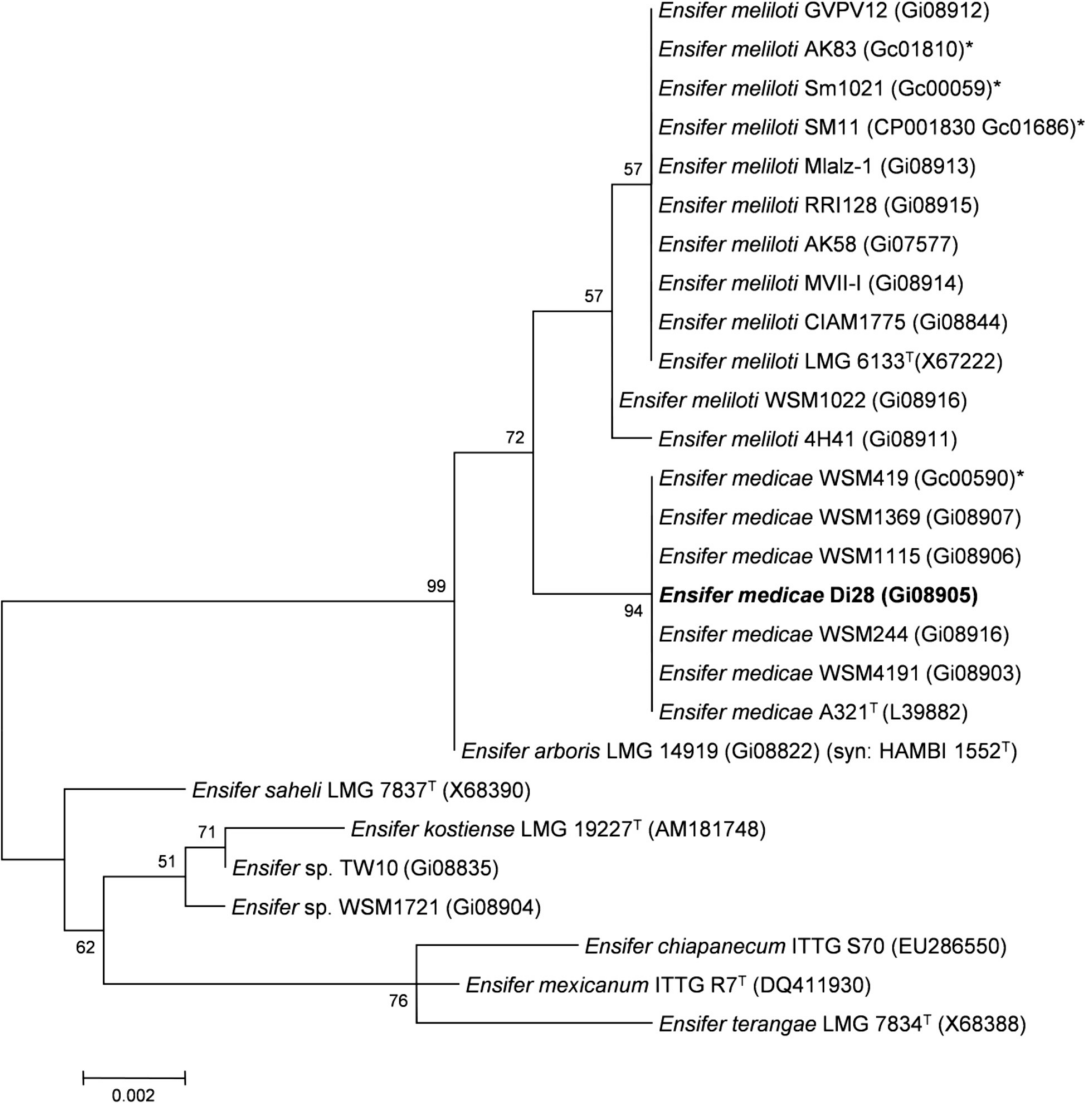
**Phylogenetic tree showing the relationship of *Ensifer medicae *Di28 (shown in bold print) to other *Ensifer *spp. in the order *Rhizobiales *based on aligned sequences of the 16S rRNA gene (1,290 bp internal region).** All sites were informative and there were no gap-containing sites. Phylogenetic analyses were performed using MEGA, version 5 [27]. The tree was built using the Maximum-Likelihood method with the General Time Reversible model [28]. Bootstrap analysis [29] with 500 replicates was performed to assess the support of the clusters. Type strains are indicated with a superscript T. Brackets after the strain name contain a DNA database accession number and/or a GOLD ID (beginning with the prefix G) for a sequencing project registered in GOLD [30]. Published genomes are indicated with an asterisk.

Minimum Information about the Genome Sequence (MIGS) is provided in Table 1. Figure 2 shows the phylogenetic neighborhood of *E. medicae* Di28 in a 16S rRNA sequence based tree. This strain shares 100% sequence identity (over 1,290 bp) to the 16S rRNA of the *E. medicae* A321 type strain and the fully sequenced *E. medicae* WSM419 [11].

**Table 1:** **Classification and general features of *Ensifer medicae *** **Di28 according to the MIGS recommendations **[15]

**MIGS ID**	**Property**	**Term**	**Evidence code**
	Current classification	Domain *Bacteria*	TAS [16]
		Phylum *Proteobacteria*	TAS [17]
		Class *Alphaproteobacteria*	TAS [18,19]
		Order *Rhizobiales*	TAS [19,20]
		Family *Rhizobiaceae*	TAS [21,22]
		Genus *Ensifer*	TAS [23-25]
		Species *Ensifer medicae*	TAS [24]
		Strain Di28	
	Gram stain	Negative	IDA
	Cell shape	Rod	IDA
	Motility	Motile	IDA
	Sporulation	Non-sporulating	NAS
	Temperature range	Mesophile	NAS
	Optimum temperature	28°C	IDA
	Salinity	Non-halophile	NAS
MIGS-22	Oxygen requirement	Aerobic	TAS [8]
	Carbon source	Varied	NAS
	Energy source	Chemoorganotroph	NAS
MIGS-6	Habitat	Soil, root nodule, on host	NAS
MIGS-15	Biotic relationship	Free living, symbiotic	TAS [8]
MIGS-14	Pathogenicity	Non-pathogenic	NAS
	Biosafety level	1	TAS [26]
	Isolation	Root nodule	TAS [10]
MIGS-4	Geographic location	Sardinia, Italy	TAS [10]
MIGS-5	Soil collection date	March-May 1998	TAS [10]
MIGS-4.1	Longitude	9.517034	TAS [10]
MIGS-4.2	Latitude	39.11260	TAS [10]
MIGS-4.3	Depth	0-10 cm	TAS [10]
MIGS-4.4	Altitude	10 m above sea level	TAS [10]

Evidence codes – IDA: Inferred from Direct Assay; TAS: Traceable Author Statement (i.e., a direct report exists in the literature); NAS: Non-traceable Author Statement (i.e., not directly observed for the living, isolated sample, but based on a generally accepted property for the species, or anecdotal evidence). These evidence codes are from the Gene Ontology project [31].

### Symbiotaxonomy

*E. medicae* strain Di28 was isolated during a germplasm collection carried out in 1998 [10] from a nodule collected from the annual *M. polymorpha* growing near Villasimius, South East Sardinia (Italy). The site of collection contained ruderal plant species, with soil properties of 1.69% (w/w) organic matter, 0.09% (w/w) total nitrogen and a near-neutral pH. Along with *M. polymorpha*, other *Medicago* spp. present at the sampling site were *M. rugosa*, *M. littoralis* and *M. rigidula*. Di28 forms nodules and fixes N_2_ with *M. sativa*, *M. polymorpha* and *M. murex*. However, while Di28 is fully effective for N_2_ fixation with *M. murex* and *M. polymorpha*, it is only partially effective as a microsymbiont of *M. sativa*.

## Genome sequencing and annotation information

### Genome project history

This organism was selected for sequencing on the basis of its environmental and agricultural relevance to issues in global carbon cycling, alternative energy production, and biogeochemical importance, and is part of the Community Sequencing Program at the U.S. Department of Energy, Joint Genome Institute (JGI) for projects of relevance to agency missions. The genome project is deposited in the Genomes OnLine Database [30] and a standard draft genome sequence in IMG. Sequencing, finishing and annotation were performed by the JGI. A summary of the project information is shown in Table 2.

**Table 2:** **Genome sequencing project information for *E. medicae *** **Di28**

**MIGS ID**	**Property**	**Term**
MIGS-31	Finishing quality	Standard draft
MIGS-28	Libraries used	One Illumina fragment library
MIGS-29	Sequencing platforms	Illumina HiSeq 2000
MIGS-31.2	Sequencing coverage	Illumina: 374×
MIGS-30	Assemblers	Velvet version 1.1.04; Allpaths-LG version r39750
MIGS-32	Gene calling methods	Prodigal 1.4
	Genbank accession	ATTL00000000
	Genbank Registration Date	December 12, 2013
	GOLD ID	Gi08905
	NCBI project ID	162987
	Database: IMG	2513237089
	Project relevance	Symbiotic N_2_ fixation, agriculture

### Growth conditions and genomic DNA preparation

*E. medicae* Di28 was cultured to mid logarithmic phase in 60 ml of TY rich media [32] on a gyratory shaker at 28°C at 250 rpm. DNA was isolated from the cells using a CTAB (Cetyl trimethyl ammonium bromide) bacterial genomic DNA isolation method [33].

### Genome sequencing and assembly

The genome of *Ensifer medicae* Di28 was sequenced at the Joint Genome Institute (JGI) using Illumina technology [34]. An Illumina standard paired-end library was constructed and sequenced using the Illumina HiSeq 2000 platform which generated 16,333,536 reads totaling 2,450 Mbp.

All general aspects of library construction and sequencing performed at the JGI can be found at DOE Joint Genome Institute user homepage. All raw Illumina sequence data was passed through DUK, a filtering program developed at JGI, which removes known Illumina sequencing and library preparation artifacts (Mingkun, L., Copeland, A. and Han, J., unpublished). The following steps were then performed for assembly: (1) filtered Illumina reads were assembled using Velvet [35] (version 1.1.04), (2) 1–3 Kb simulated paired end reads were created from Velvet contigs using wgsim [36], (3) Illumina reads were assembled with simulated read pairs using Allpaths–LG [37] (version r39750). Parameters for assembly steps were: 1) Velvet (velveth: 63 –shortPaired and velvetg: -veryclean yes –exportFiltered yes –mincontiglgth 500 –scaffolding no–covcutoff 10) 2) wgsim (-e 0–1 76–2 76 -r 0 -R 0 -X 0) 3) Allpaths–LG (PrepareAllpathsInputs:PHRED64 = 1 PLOIDY = 1 FRAGCOVERAGE = 125 JUMPCOVERAGE = 25 LONGJUMPCOV = 50, RunAllpath-sLG: THREADS = 8 RUN = stdshredpairs TARGETS = standard VAPIWARNONLY = True OVERWRITE = True). The final draft assembly contained 104 contigs in 104 scaffolds. The total size of the genome is 6.5 Mbp and the final assembly is based on 2,450 Mbp of Illumina data, which provides an average 374× coverage of the genome.

### Genome annotation

Genes were identified using Prodigal [38] as part of the DOE-JGI annotation pipeline [39]. The predicted CDSs were translated and used to search the National Center for Biotechnology Information (NCBI) nonredundant database, UniProt, TIGRFam, Pfam, PRIAM, KEGG, COG, and InterPro databases. The tRNAScanSE tool [40] was used to find tRNA genes, whereas ribosomal RNA genes were found by searches against models of the ribosomal RNA genes built from SILVA [41]. Other non–coding RNAs such as the RNA components of the protein secretion complex and the RNase P were identified by searching the genome for the corresponding Rfam profiles using INFERNAL [42]. Additional gene prediction analysis and manual functional annotation was performed within the Integrated Microbial Genomes (IMG-ER) platform [43].

## Genome properties

**Figure 3:**
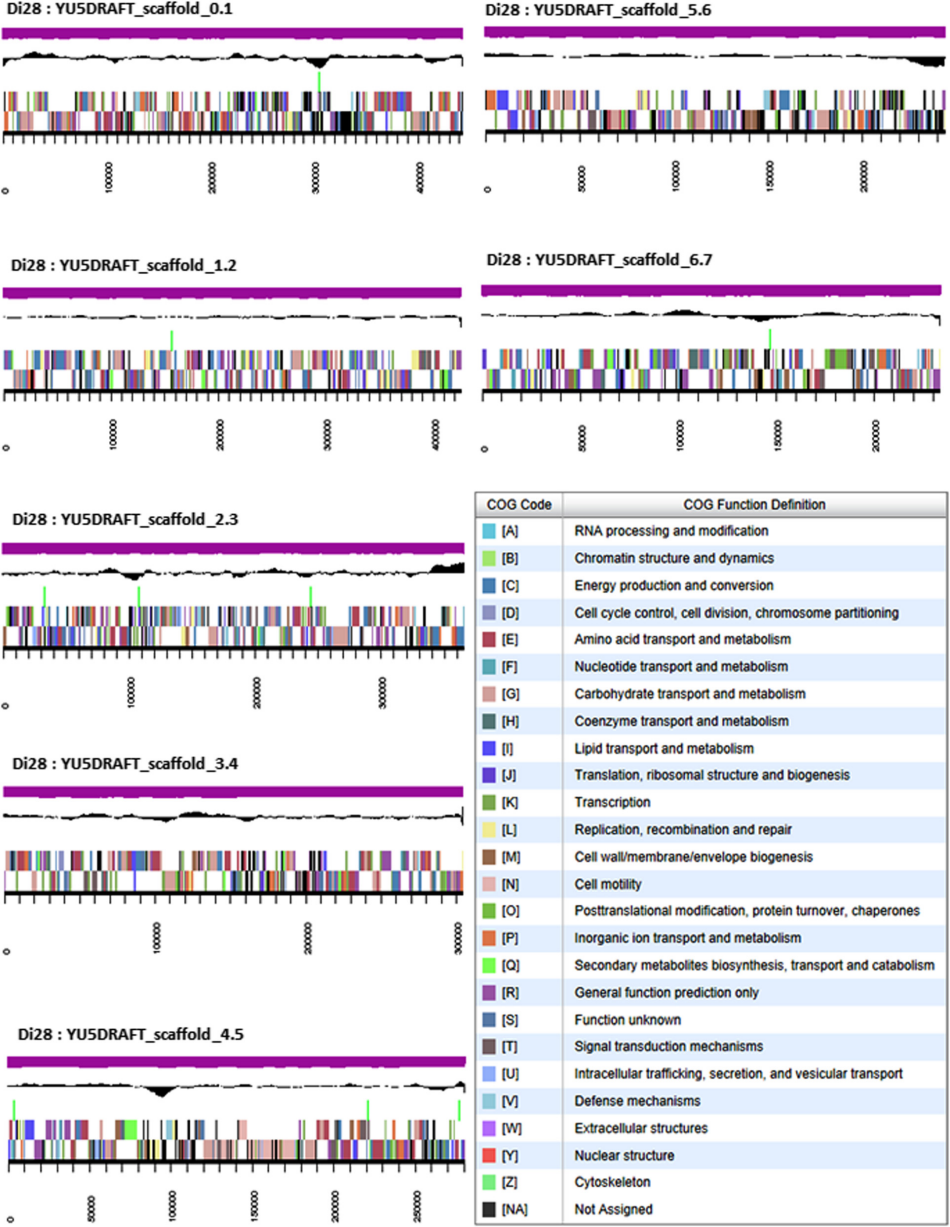
**Graphical map of the genome of *Ensifer medicae *Di28 showing the seven largest scaffolds.** From bottom to the top of each scaffold: Genes on forward strand (color by COG categories as denoted by the IMG platform), Genes on reverse strand (color by COG categories), RNA genes (tRNAs green, sRNAs red, other RNAs black), GC content, GC skew.

The genome is 6,553,624 nucleotides with 61.15% GC content (Table 3) and comprised of 104 scaffolds (Figure 3) of 104 contigs. From a total of 6,469 genes, 6,394 were protein encoding and 75 RNA only encoding genes. The majority of genes (78.65%) were assigned a putative function whilst the remaining genes were annotated as hypothetical. The distribution of genes into COGs functional categories is presented in Table 4.

**Table 3:** **Genome Statistics for *Ensifer medicae *** **Di28**

**Attribute**	**Value**	**% of total**
Genome size (bp)	6,553,624	100.00
DNA coding region (bp)	5,702,238	87.03
DNA G + C content (bp)	4,007,255	61.15
Number of scaffolds	104	
Number of contigs	104	
Total gene	6,469	100.00
RNA genes	75	1.16
rRNA operons	1	0.02
Protein-coding genes	6,394	98.84
Genes with function prediction	5,088	78.65
Genes assigned to COGs	5,052	78.10
Genes assigned Pfam domains	5,259	81.30
Genes with signal peptides	534	8.25
Genes with transmembrane helices	1,449	22.40
CRISPR repeats	0	

**Table 4:** **Number of protein coding genes of *Ensifer medicae *** **Di28 associated with the general COG functional categories**

**Code**	**Genecount**	**% age**	**Description**
J	188	3.34	Translation, ribosomal structure and biogenesis
A	0	0.00	RNA processing and modification
K	491	8.73	Transcription
L	222	3.95	Replication, recombination and repair
B	1	0.02	Chromatin structure and dynamics
D	40	0.71	Cell cycle control, mitosis and meiosis
Y	0	0.00	Nuclear structure
V	61	1.08	Defense mechanisms
T	226	4.02	Signal transduction mechanisms
M	279	4.96	Cell wall/membrane biogenesis
N	68	1.21	Cell motility
Z	0	0.00	Cytoskeleton
W	1	0.02	Extracellular structures
U	104	1.85	Intracellular trafficking and secretion
O	178	3.16	Posttranslational modification, protein turnover, chaperones
C	339	6.03	Energy production conversion
G	594	10.56	Carbohydrate transport and metabolism
E	630	11.20	Amino acid transport metabolism
F	109	1.94	Nucleotide transport and metabolism
H	192	3.41	Coenzyme transport and metabolism
I	212	3.77	Lipid transport and metabolism
P	286	5.08	Inorganic ion transport and metabolism
Q	160	2.84	Secondary metabolite biosynthesis, transport and catabolism
R	690	12.26	General function prediction only
S	555	9.86	Function unknown
-	1417	21.90	Not in COGS

## Conclusions

Di28 was isolated from a nodule of *M. polymorpha* found in Sardinian soil of near-neutral pH. The genome size, gene count, GC content and COG profile of Di28 is comparable to that of the sequenced *E. medicae* strains WSM244, WSM419, WSM1115, WSM1369 and WSM4191. Of particular interest is the finding that Di28, WSM244 and WSM1369 have a relatively low pseudogene percentage (0.03-0.06%) in comparison to the other strains (4.29-6.83%). One stand-out feature from the genome of Di28 is the absence of the acid-activated *lpiA* gene (11,32), which is found in all other *E. meliloti* and *E. medicae* strains sequenced to date. Furthermore, the regulatory genes *tcsA*, *tcrA* and *fsrR*, which are required for the full acid-activated expression of *lpiA*, are present in all other sequenced *E. medicae* strains, but are absent in Di28. The unique attributes of Di28 in comparison to other *Ensifer strains*, make this an ideal candidate for future work.

## Competing interests

The authors declare that they have no competing interests.

## Authors’ contributions

GG and JH supplied the strain and background information for this project, TR supplied DNA to JGI and performed all imaging, GG and JT drafted the paper, YH performed phylogenetic analysis, WR coordinated the project and all other authors were involved in either sequencing the genome and/or editing the paper. All authors read and approved the final manuscript.
